# Iron Overload, Oxidative Stress, and Ferroptosis in the Failing Heart and Liver

**DOI:** 10.3390/antiox10121864

**Published:** 2021-11-24

**Authors:** Daniele Mancardi, Mariarosa Mezzanotte, Elisa Arrigo, Alice Barinotti, Antonella Roetto

**Affiliations:** Department of Clinical and Biological Sciences, University of Torino, 1043 Orbassano, Italy; mariarosa.mezzanotte@unito.it (M.M.); elisa.arrigo@unito.it (E.A.); alice.barinotti@unito.it (A.B.); antonella.roetto@unito.it (A.R.)

**Keywords:** oxidative stress, heart failure, hepatic failure, iron, ferroptosis

## Abstract

Iron accumulation is a key mediator of several cytotoxic mechanisms leading to the impairment of redox homeostasis and cellular death. Iron overload is often associated with haematological diseases which require regular blood transfusion/phlebotomy, and it represents a common complication in thalassaemic patients. Major damages predominantly occur in the liver and the heart, leading to a specific form of cell death recently named ferroptosis. Different from apoptosis, necrosis, and autophagy, ferroptosis is strictly dependent on iron and reactive oxygen species, with a dysregulation of mitochondrial structure/function. Susceptibility to ferroptosis is dependent on intracellular antioxidant capacity and varies according to the different cell types. Chemotherapy-induced cardiotoxicity has been proven to be mediated predominantly by iron accumulation and ferroptosis, whereas there is evidence about the role of ferritin in protecting cardiomyocytes from ferroptosis and consequent heart failure. Another paradigmatic organ for transfusion-associated complication due to iron overload is the liver, in which the role of ferroptosis is yet to be elucidated. Some studies report a role of ferroptosis in the initiation of hepatic inflammation processes while others provide evidence about an involvement in several pathologies including immune-related hepatitis and acute liver failure. In this manuscript, we aim to review the literature to address putative common features between the response to ferroptosis in the heart and liver. A better comprehension of (dys)similarities is pivotal for the development of future therapeutic strategies that can be designed to specifically target this type of cell death in an attempt to minimize iron-overload effects in specific organs.

## 1. Introduction

**Figure 1:**
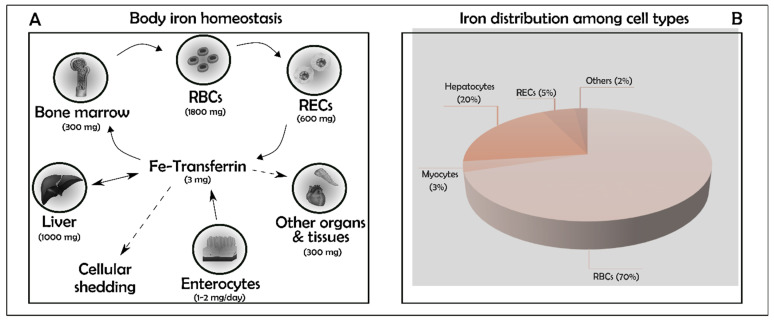
(**A**,**B**) Iron homeostasis and distribution. Summary of the main iron accumulation sites in the human body. In parenthesis the amount of the metal in each compartment. Iron trafficking is a dynamic process: transferrin is used to transport iron. Total iron together with myocytes (3%) and other minor types (2%) cover just one tenth of the total. Major contribution to iron storage capacity comes from Red Blood Cells (RBCs, 70%) and hepatocytes (20%). Macrophages remove senescent erythrocytes, clear haemoglobin-derived heme via hemeoxygenases (HO-1 and HO-2), and export the remaining Fe^2+^ to plasma via Ferroportin (Fpn1), the sole cellular iron exporter. Hepatocytes store excess of body iron within ferritin, the iron storage protein. RBCs: Red Blood Cells; RECs: Reticuloendothelial Cells.

Iron is a pivotal element for cell metabolism and a key regulator of several cellular functions mainly through enzymatic activity modulation. The biological importance of iron is largely attributable to its chemical properties; as an element belonging to the transition metals, it promptly undertakes oxidation/reduction reactions between its ferric-reduced (Fe^3+^) and ferrous-oxidized (Fe^2+^) states. Iron is an essential component of haemoproteins such as myoglobin, haemoglobin, cytochrome p450, iron–sulphur proteins, and several other proteins that are involved in many aspects of cellular metabolism [1]. Under physiological conditions, there is no specific mechanism for iron excretion from the body, and iron metabolism is strictly influenced by its absorption through the diet (1–2 mg Fe/day), to compensate for nonspecific losses of the metal (mainly through cellular desquamation, menstrual bleeding, or other occasional blood loss) (Figure 1). Iron trafficking is a dynamic process: Transferrin mediates the transport among sites for absorption, recycling, storage, and utilization. Iron released by duodenal enterocytes and splenic macrophages is transferred to bone marrow where it is used to produce RBCs and to the liver where it is stored (Figure 1).

Iron absorption is controlled via a negative feedback regulatory mechanism, that involves the iron-regulator Hepcidin (Hepc) [2]. In some organs, including the heart, liver, duodenum and bone marrow, iron metabolism is finely regulated.

## 2. Iron Balance

The adult human body content of iron is approximately 3–5 g, corresponding to ~55 mg/kg in males and ~44 mg/kg in females [3,4], differently distributed according to cell type (Figure 1B).

**Figure 2:**
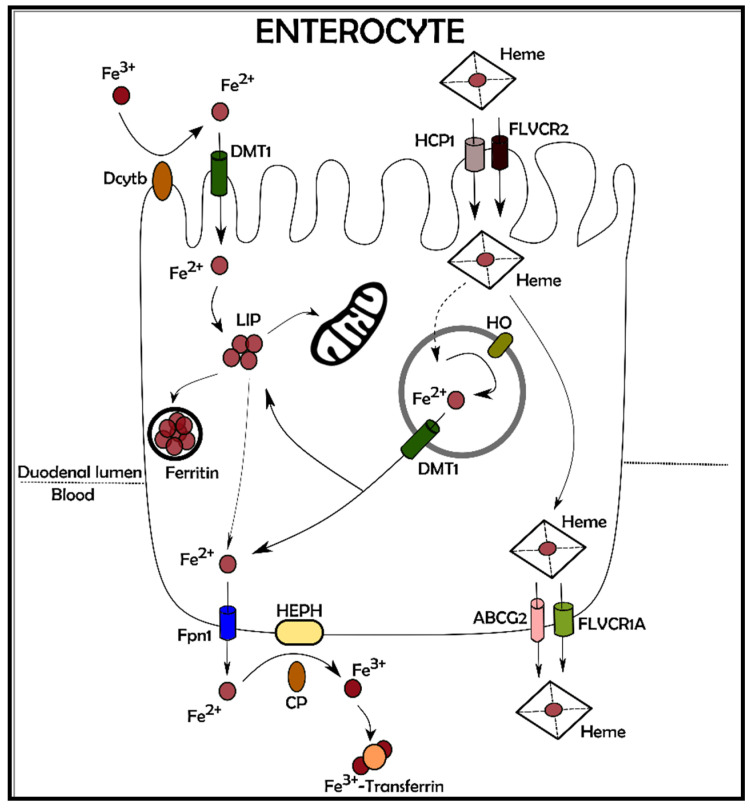
Dietary iron absorption. The iron ingested with the diet can be inorganic (ferric iron, Fe^3+^) or incorporated in heme. Ferric iron is converted in its ferrous state (Fe^2+^) by Dcytb, a ferric reductase enzyme on the brush border of the enterocytes. Fe^2+^ is then transported within the cell through DMT1, a transporter placed in the apical membrane of the enterocytes. Once inside the cell, the absorbed inorganic iron can be used for immediate biologic processes, stored in ferritin or released into circulation through Fpn1, the main iron exporter positioned in the basolateral membrane of enterocytes. To be bound to transferrin, Fe^2+^ must be re-oxidized in its ferric state by HEPH or CP. Intestinal heme is possibly incorporated through HCP1 or FLVCR2 transporters. Within the enterocyte, heme can be released unmodified into circulation or it can be degraded in endosomes/lysosomes by HO. The liberated iron follows the same fate of the absorbed inorganic iron. Dcytb: Duodenal cytochrome b; DMT1: Divalent Metal Transporter 1; LIP: Labile Iron Pool; Fpn1: Ferroportin 1; HEPH: Hephaestin; CP: Ceruloplasmin; ABCG2: ATP-Binding Cassette Subfamily G Member 2; FLVCR1A: Feline Leukaemia Virus subgroup C cellular Receptor 1A; HO: Heme Oxigenase; HCP1: Heme Carrier Protein 1; FLVCR2: Feline Leukaemia Virus subgroup C cellular Receptor 2.

Nutritional iron is absorbed by two main sources: inorganic iron and heme iron, derived from red meat, this last having a higher bioavailability. The pathways involved in heme iron metabolism have been recently elucidated with a deeper understanding on the role of heme importers and exporters mediating the trafficking through the plasma membrane [5]. Similarly, the mechanism leading to inorganic iron absorption is quite well described [6], involving reduction of Fe^3+^ to Fe^2+^ by ferric reductases (such as Dcytb) or other reducing agents (such as ascorbate) in the small intestine lumen. Absorption is facilitated by transport across the apical membrane of enterocytes via the Divalent Metal Transporter 1 (DMT1). Internalized Fe^2+^ is subsequently transferred to the basolateral membrane by a rather unknown mechanism and exported to interstitial fluid and plasma via Ferroportin 1 (Fpn1). The externalization of Fe^2+^ is associated with its re-oxidation to Fe^3+^ by either the soluble or membrane-bound multicopper ferroxidases ceruloplasmin or hephaestin, respectively (Figure 2) [7].

The highest amount of iron is consumed by erythropoiesis although it is largely recycled from phagocytosis of senescent RBCs, mainly by splenic reticuloendothelial macrophages. The modulation of erythropoiesis is, in turn, adjusted by a pleiotropic regulator of iron metabolism, Hepcidin (Hepc, described below) together with Hepc newly described modulators (Gdf15, Twgs1, Erfe) although the precise mechanism of action has not yet been completely clarified [8].

Myocytes are the second major site of iron utilization, used to produce myoglobin [9] even though the mechanism of iron incorporation for myoglobin production is not well described. On the other hand, hepatocytes are the principal reservoirs for iron deposition and storage, but when the body content of iron is re-established, macrophages of the liver, spleen, and bone marrow also contribute to storing it. The iron amount in hepatocytes and macrophages can be mobilized to meet erythropoietic and cellular demands when body iron levels are low. An active mechanism for the excretion of iron is missing and only a very small amount is present each day in the urine or faeces [10]. Iron is also lost in females with blood during menstruation and childbirth, however iron loss from sweat and desquamated skin cells is negligible even during intense physical exercise [11,12]. As mentioned before, most of the iron in the human organism is retrieved from senescent red blood cells through the RECs of the spleen and the Kupffer cells of the liver. 

## 3. Physiopathology Iron-Related in Heart and Liver 

**Figure 3:**
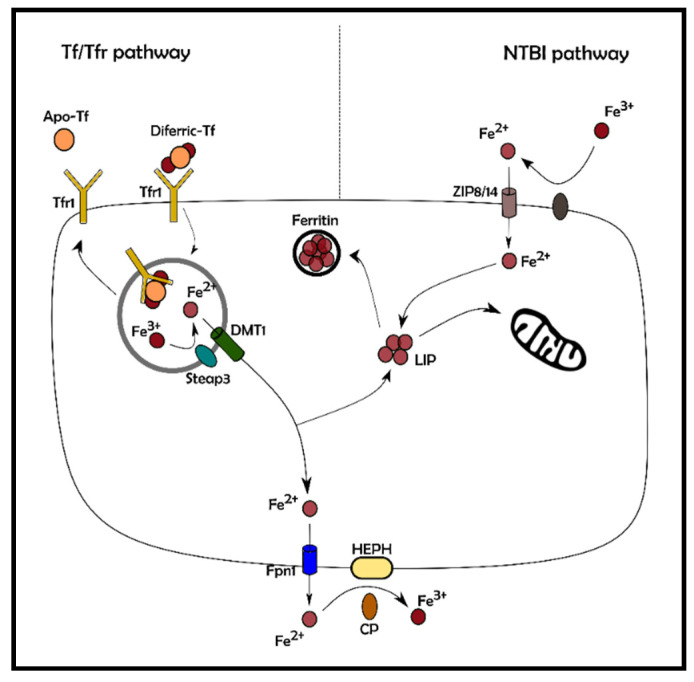
Non-heme iron absorption. Iron can be absorbed by the cells following two pathways: Transferrin/Transferrin Receptor 1 (Tf/Tfr1, on the left) or non-transferrin-bound iron (NTBI, on the right). The blood circulating diferric transferrin (diferric-Tf) binds to Tfr1, a membrane-bound receptor. The complex is transferred within the cells into endosomes; here, the acidic pH generated by proton pumps, results in the release of the ferric iron from the complex. Fe^3+^ is reduced in its ferrous state by Steap3 and released into the cytosol through DMT1. The Tf/Tfr1 complex is recycled, while the free cytosolic ferrous iron follows the same fate as mentioned in Figure 2. The uptake of the NTBI pool seizes on carriers such as DMT1 or the most recently studied zinc transporters ZIP8 and ZIP14. NTBI: Non-Transferrin-Bound Iron; Tf: Transferrin; Tfr1: Transferrin receptor 1; DMT1: Divalent Metal Transporter 1; LIP: Labile Iron Pool; Fpn1: Ferroportin 1; Steap3: Six-transmembrane epithelial antigen of prostate 3; HEPH: Hephaestin; CP: Ceruloplasmin; ZIP8: Zrt–Irt-like Protein 8; ZIP14: Zrt–Irt-like Protein 14, Apo-TF: iron-free Tf.

In several clinical scenarios, including primary hemochromatosis and secondary iron-overload, iron metabolism is chronically altered causing, in combination with confounding environmental factors, increased morbidity and mortality. Most interestingly, patients affected by iron-overload associated diseases (i.e., hemoglobinopathy and hemochromatosis) can manifest signs of organ dysfunction often degenerating in liver and heart failure. In iron-overload conditions, serum levels of iron are typically higher than the transferrin iron-binding capacity, leading to the increase of the content of iron non-transferrin-bound, which has a higher reactivity. It is well established that iron can enter the cells by two main mechanisms: either bound to the transporter protein Transferrin (Tf), through Transferrin/Transferrin receptor 1 (Tfr1) or, in form of non-transferrin-bound iron (NTBI), through Divalent Metal Ionic Transporter (DMT) which is associated with metallo-reductases Dcytb and the STEAP3 proteins [13]. Recently, new players have been shown to be involved in iron uptake, namely the neutral pH transporters ZIP8 and ZIP14 as well as a group of cytochrome-dependent oxido-reductases that shuttle reducing equivalents across the plasma membrane according to the plasma membrane electron transport (PMET) mechanism [13]. On the contrary, iron export from cells occurs through Fpn1, associated with different ferroxidases (ceruloplasmin, hephaestin, and zyclopen (HEPHL)) (Figure 3) [14]. Excessive NTBI uptake, along with ineffective iron excretory pathways, lead to a significant increase of the so-called labile intracellular iron pool (LIP), as well as the formation of highly reactive oxygen free radicals, augmenting detrimental peroxidation of membrane lipids and diffuse oxidative damage to cellular proteins [15]. Due to its potential toxicity (see above), intracellular iron is managed to allow a minimal amount of free iron within the cytoplasm. For this reason, it is essentially associated with the iron deposit protein, Ferritin (Ft) or utilized in cytoplasmic and mitochondrial iron requiring proteins. Mitochondria, in particular, use a consistent amount of cellular iron as a cofactor in several proteins involved in oxidation/reduction reactions of the respiratory chain [16].

Cellular iron homeostasis is regulated post transcriptionally by the so-called IRE/IRP system that involves the iron regulatory protein 1 (IRP1) and 2 (IRP2) (also known as ACO1 and IREB2, respectively) and two iron–sulphur (Fe–S) RNA-binding proteins that are able to bind to a *cis*-regulatory hairpin structures known as IRE (Iron Regulatory Element), located in the 5′ or 3′ untranslated regions (UTRs) of target mRNAs. In the condition of cellular iron deprivation, either of the two IRPs binds to the 5′ UTR IREs of target RNA, impeding the transcription initiation complex to exert its function and inhibiting the RNAs translation [17]. On the contrary, IRPs binding to the 3’ UTR IRE motif elements present in specific mRNAs prevent their endonucleolytic cleavage and subsequent degradation [18]. Consistently, other studies report the involvement of other elements of the IRE/IRP regulatory network including the human cell division cycle 14A mRNA [19] and human MRCKalpha [20] (See Table 1).

## 4. Iron Metabolism in the Heart

Cardiac myocytes are major oxygen consumers, and a commensurate intracellular iron pool is necessary to sustain this aerobic activity. The high metabolic demand of the heart and its dependency on iron is confirmed by the finding about the deletion of heart Tfr1 in a murine model which causes detrimental energetic failure in cardiac myocytes, even in the presence of normal systemic iron levels [21]. Normally, cardiomyocytes have relatively high levels of Hepc and Fpn1, although they have not been proved essential in controlling systemic iron accumulation and metabolism [22,23]. Most recently, the cardiac role of Hepc and Fpn1 has been investigated with animal models characterized by cardiomyocyte-specific downregulation of the Hepc/Fpn1 axis. Interestingly, cardiac specific deletion of Fpn1 encoding gene prompted mice to develop fatal dysfunction of the left ventricle by the third month of age. The dysfunction was associated with a threefold increase in iron levels within cardiac myocytes. It is worthy of note, that Tfr1 downregulation does not counteract ferroportin 1 deficiency to prevent iron overload in these hearts, indicating that iron release, mediated by ferroportin 1, is essential for iron homeostasis in the heart, along with other iron intake mechanism(s) [23]. In the same model of Fpn1 deficient heart, iron was predominantly stored in the cardiomyocytes, whilst in the hemochromatosis murine model, a major part of the iron was in the extracellular compartment, consistent with a significant increase in cardiomyocyte Fpn1. Therefore, ferroportin 1 is not only crucial for iron homeostasis in the heart, but it also responsible for deposition of iron in the heart in the conditions characterized by systemic iron overload. In fact, ferroportin 1 may be held responsible for the severity of cardiac dysfunction determined by iron deposition [23]. On the other side, specific deletion of Hepc in the heart of mice leads to a fatal dysfunction of the left ventricle between 3 and 6 months of age, even if systemic iron levels are maintained in a normal range. All these results together, suggest that the myocardial hepcidin/ferroportin 1 pathway is pivotal for the autonomous control of the intracellular iron stores which are required for physiological cardiomyocytes functions. The same cardiac hepcidin/ferroportin 1 axis has been also proven to be important to protect the myocardium from the consequences of systemic iron deficiency. Cardiac levels of Hepc protein were increased instead of decreased after dietary iron restriction in in vivo models and by iron chelation in in vitro experiments. Moreover, animal models with cardiac depletion of hepcidin have increased chances to develop hypertrophy in response to a sustained dietary iron restriction when compared to their littermate controls [22].

Although all cell types forming the heart are sensible to injury triggered by increased ROS production, cardiomyocytes have been shown to be particularly affected [23,24]. The high metabolic demand of the myocardium, in fact, is matched by a peculiar abundance in mitochondria with a consequent elevated oxygen consumption and a detrimental deficiency in antioxidant enzymes [25]. Unbalance of cardiomyocytes iron content is regulated by the expression of Tfr1 [26] and iron overload becomes critical when NTBI/LPI is exceeded due to the saturated binding capacity of Tf [27]. The exact mechanism for NTBI to enter cardiomyocytes is still largely undescribed although some studies report an involvement of L-type or T-type calcium channels in iron uptake under overload conditions and suggesting calcium channel blockers as a potential therapeutic tool to mitigate cardiac effects of iron overload [15]. This evidence is supported by ex vivo studies which demonstrated that L-type Ca^2+^ channels downregulation can reduce iron uptake under chronic overload condition [28]. In case of iron overload, therefore, L-type channels mediate the increased ferrous iron uptake which leads to an over production of ROS that triggers, among others, an excitation-contraction uncoupling with an impaired systolic and diastolic function, a typical sign of iron-overload cardiomyopathy [29]. Another iron carrier has been proposed to be potentially involved in the developing of heart failure, lipocalin-2 [30]. It has been reported that lipocalin-2 induces apoptosis in in vitro settings while the reported in vivo effects include acute inflammatory response and cardiac remodelling [31].

On the other hand, for the oxygen to react with intracellular molecules a catalyst is needed, one of the most potent being iron, and when LIP increases, oxidative stress can pose a cytotoxic trigger mediated by accelerating the reaction between oxygen and biomolecules. Typically, the increased ferritin expression leads to a safe cytosolic stored iron as a part of the response to a pro-oxidative environment [32]. 

Most interestingly, oxidative stress in the heart plays a double role as a mediator of both protection and generation of the ischemia/reperfusion injury [33]. In addition, cardiac ferritin is enriched in H subunit [25], undergoing an iron-based mechanism in the ischemic preconditioning phenomenon [34]. In the regulation of intracellular iron, exporting excess could represent a way to counterbalance an exceeding entry of NTBI. Basal expression of H-ferritin cannot cope with iron overload and regulation of its expression is necessary for a rapid chelation of LIP to prevent oxidative stress [35] and these findings suggest a putative role for transcriptional regulation of iron transporters and ferritin subunits in the heart [36]. Paradoxically, Hepc levels can be decreased in β thalassemia-mediated cardiomyopathy as well as other iron overload-mediated conditions, where erythropoiesis-dependent downregulation of Hepc should prevail and these low levels of circulating Hepc are believed partially responsible for the ferroportin 1 expression and export activity, thereby concurring to limit iron accumulation in the heart [25].

## 5. Iron and Oxidative Stress in Cardiomyocytes and Hepatocytes

While it seems clear that iron externalization is almost solely regulated by ferroportin 1, the degree of internalization is synergistically determined by several transporters, some of which are tissue-specific and others are ubiquitous.

The estimated iron concentrations in the major cellular compartments (~6 μM cytosol [37], ~16 μM mitochondria [38], ~7 μM nuclei [39], and ~16 μM lysosomes [39]) are finely regulated by specific transporters. Under excessive extracellular iron, cardiac cells can uptake Fe^3+^ or Fe^2+^ through L-type and T-type Ca^2+^ channels [40], although in some models verapamil and amlodipine, but not efonidipine (all calcium-channel blockers), could mitigate iron uptake. In mitochondria, where iron is critical for several reasons, including the production of heme- and iron–sulphur-cluster proteins of the electron transport chain, a putative role has been shown for the calcium uniporter: blocking the activity of this specific transporter, leads to the preservation of mitochondrial structure and function after iron overload of cardiomyocytes [41]. On the other hand, two nuclear-encoded proteins, Mitoferrin1 (Mfrn1) and Mitoferrin2 (Mfrn2) have been shown to mediate internalization of iron [42] suggesting a multifactorial regulation of iron levels in mitochondria. Genetic deletion of Mfrn1 induces severe impairment of heme synthesis and is lethal during early embryonic development while its transcription, promoted by GATA1, is upregulated during erythropoiesis [43]. When Mfrn1 expression is experimentally blunted during adulthood, the phenotype is vital with defects in erythroid cells resulting in severe anaemia [44]. Most interestingly, mRNA for Mfrn1 is detectable in non-erythropoietic cell lines with lower levels compared to Mfrn2, which is ubiquitously expressed in virtually all tissues [43]; nevertheless, Mfrn1 absence is not shown to affect the development of heart, liver, or muscle [44]. With its ability to shift between two redox states, non-bound iron is highly reactive, leading to oxidative stress which is a pivotal trigger of tissue and organ failure [15]. The key step for the excessive production of radicals is the Fenton reaction, which has been demonstrated to play a fundamental role in the physiopathology of several diseases, including heart failure [45], liver failure [46], renal disease [47], and different types of neurodegeneration [48,49,50]. Cardiomyopathies related to iron-overload represent one of the major causes of mortality and comorbidity in patients with secondary and primary hemochromatosis, respectively [15,51,52,53]. Aside from direct ROS-mediated damage, iron overload has been shown to impair synthesis and function of respiratory subunits through mitochondrial DNA (mtDNA) alteration [54]. Full length mtDNA is accessed to verify the integrity of the mitochondrial genome and it is substantially reduced by 300 μm of extracellular iron [54].

## 6. Ferroptosis in Heart Failure

Numerous cardiovascular diseases are characterized by cardiac and vascular cell loss trough necrosis, apoptosis, and autophagy. As a novel type cell death, ferroptosis has been reported to play a pivotal role in the onset and development of various cardiovascular diseases mediated by an accumulation of lethal lipid hydroperoxides triggered by iron [55]. In the heart, several mechanisms are critical for the triggering of ferroptosis, including iron metabolism, glutamine metabolism, and lipid peroxidation, while recent studies suggest that myocardial dysfunction induced by ferroptosis can be inhibited by iron chelators and antioxidants [56]. In hemochromatosis patients, there is a significant deterioration of electro-mechanical coupling of cardiomyocytes mediated by ROS formation, while major cardiotoxic effects of doxorubicin have been proven to be ferroptosis-mediated [57]. Most interestingly, doxorubicin cytotoxicity on myocytes can be decreased through inhibition of ferroptosis induced by over expression of GPX4 and activation of Keap1 (Kelch-like ECH-associated protein 1)/Nrf2 signalling [57]. The involvement of ferroptosis in chemotherapy-induced cardiomyopathy, is confirmed by transgenic mouse models in which sequestration of Keap1 grants cardiac protection from doxorubicin-induced ferroptosis [58]. Consistently, other reports provide evidences about the role of Acyl-CoA thioesterase in preventing myocardial injury in doxorubicin-treated murine hearts [59].

Being characterized by the death of post-mitotic cardiomyocytes, heart failure (HF) is an irreversible condition for which an early prevention of cell loss is of paramount importance. Independently of the type of cell death to be targeted, retarding progression of heart failure is fundamental to preserve cardiac function as shown by a report describing a ferroptotic pathway in an animal model of HF induced by left ventricular pressure overload [60]. The interplay between HF and iron metabolism is complex and rich in negative feedback mechanisms leading to a general worsening of patient conditions. Anaemia, for instance, is often found in HF patients and prognosis is generally poorer according to blood haemoglobin content [61]. Among the pathogenic mechanisms linking anaemia and HF there is iron deficiency, although it is not clear which one may be held primarily responsible for the vicious loop [62] despite the abundance of reports describing iron-deficiency anaemia in patients with severe HF [63]. Some insights come from experimental models of HF in which a down-regulated duodenal iron transporter leads to iron deficiency and impaired liver expression of hepcidin [64]. In this study, the same outcomes in terms of iron-transporters and hepcidin expression, were achieved through an iron-deficient diet indicating that a diminished iron absorption is present in HF models [64]. Experimental data, therefore, suggest that iron supplementation may offer a clinical advantage in treating patients with HF and anaemia and three clinical trials have proven the beneficial outcomes in terms of exercise capacity in HF patients but failed to ameliorate HF-iron deficient patients [65]. The answer to these conflicting results may rely on the animal model, demonstrating a reduced absorption of iron in HF and the consequent inefficacy of oral supplementation. Consequently, a later large clinical trial investigated the effects of iron supplementation given intravenously but results and long-term safety for i.v. supplementation still need clarification [66].

There is evidence that Transferrin Receptor 2 (TfR2), a protein that plays a role in iron metabolism in different tissues [67], may be involved in erythroid iron trafficking through a mediation of lysosomes and a subsequent transfer to mitochondria [68]. Others suggest that the release of Fe^2+^ from lysosomes is mediated by Transient Receptor Potential Mucolipin 1 (TRPML1) and the mechanism can be considered, at least in part, responsible for haematological and degenerative symptoms in mucolipidosis type IV disease patients [69]. Once Fe^2+^ has reached the mitochondria, some sort of feedback is generated between the mitochondria and cytosol compartments although, when heme synthesis is impaired, iron continues to be incorporated by mitochondria probably due to poor uncoupling which leads to a positive feedback to restore heme synthesis [70].

A small mitochondrial protein, Frataxin (FXN) is involved in iron–sulphur clusters (Fe–S) formation essential for the mitochondrial respiratory chain complexes, aconitase and other mitochondrial enzymes [71]. FXN is mostly expressed in organs with high metabolic demand as liver, kidney, neurons, and heart [72]. FXN functions can be diverse and act as: (i) iron chaperone during cellular heme production [73]; (ii) iron storage protein [74]; (iii) buffer of oxidative damage directed to aconitase Fe–S clusters; (iv) protective factor against oxidative damage [75]. FXN gene mutation has been shown to be responsible for the onset of an inherited autosomal recessive neurodegenerative disorder known as Friedreich’s ataxia disease (FRDA) [76]. FRDA is characterized by mitochondrial dysfunction [77], iron accumulation in mitochondria, ROS accumulation and lipid peroxidation [78]. All these metabolic alterations draw a connection line between this disease and ferroptosis. The heart is the main organ affected by mitochondrial alteration [79] and is important to note that patients with this neurological syndrome develop a progressive hypertrophic cardiomyopathy [80]. Since the constitutive inactivation of frataxin causes embryonic lethality in mice [81] a specific striated muscle frataxin-deficient murine model and a neuron/cardiac muscle frataxin-deficient line were generated [82]. This model recapitulates the pathophysiological and biochemical peculiarity of the human disease.

## 7. The Role of Ferroptosis in Liver Failure

Several physiopathological pathways concur in the development of hepatocytes dysfunction and may lead, in the long term, to chronic liver failure. Being the second largest storage site in the body after RBCs (Figure 1), hepatocytes are constantly battling to maintain iron homeostasis and ROS balance [83]. While ROS overproduction is a well-established inducer of hepatotoxicity, only recently has the substantial contribution of ferroptosis been recognised among the other types of cell death. Indeed, most triggers commonly involved in liver failure are mediated by iron overload and consequent cell death [84]. In the drug-induced liver disease scenario, a recent study reports a prominent role of ferroptosis in a murine model of acute liver failure, induced with an overdose of acetaminophen (APAP), suggesting that this type of cell loss may represent a putative therapeutic target [85] whereas other authors disagree on the importance of the ferroptotic mechanism in this model [86]. There is consensus, however, regarding the involvement of the inhibition of ferroptosis in sorafenib resistance in the treatment of hepatocellular carcinoma [87] with a recognised role for ACSL4 (acyl-CoA synthetase long-chain family member 4), a known mediator of ferroptosis [88]. ACSL4 has been pharmacologically targeted in a mouse model of ferroptosis with a class of antidiabetics (thiazolidinediones), unveiling its role as inhibitor of ferroptosis, posing a potential therapeutic target [89]. Hepatic iron overload is often associated with hemochromatosis and intriguing results demonstrate that solute carrier family 7, member 11 (Slc7a11), an established ferroptosis-related gene, is up-regulated in iron-overloaded cells, again pointing at ferroptosis as a potential target for the treatment of hemochromatosis-induced tissue injury [90]. Moreover, in alcoholic liver steatosis and disease, ferroptosis has been proven to be increased through the deacetylase SIRT1 in mice with ethanol-induced liver injury [91] as well as in the progression of non-alcoholic steatohepatitis [92]. Non-alcoholic fatty liver disease, of which the aetiology remains largely unclear, is often associated with a high serum level of ferritin [93] and ROS-triggered ferroptosis which can be inhibited by the Keap1/Nrf2 signalling cascade [94].

Paradoxically, also when liver disease requires organ transplantation, ferroptosis must be taken into consideration, because of its role in the ischemia/reperfusion injury related to surgical procedures [95]. Not surprisingly, ACSL4 is also activated during intestinal ischemia/reperfusion and contributes to the development of ferroptosis-mediated cell loss [96] and similar mechanisms have been described in renal failure [97,98], cardiomyopathy [99], as well as in heart transplants [100].

Concluding, over the last decade it has become clearer how iron-induced toxicity is at the root of several physiopathological mechanisms and ferroptosis can, therefore, be considered a valid therapeutic target to mitigate liver failure. However, the precise molecular mediators have been identified in few cases, as for Glutathione Peroxidase 4 (GPX4) [101] and the cystine–glutamate antiporter system [102]. As a consequence, most of the data are from basic research studies whilst randomized clinical trials are still attempting to confirm results from in vivo and in vitro models.

## 8. What Is behind the Ferroptosis *Scenario*

According to recent findings, the main mechanism behind ferroptosis is ROS-dependent regulated cell death. Ferroptosis is triggered by intracellular iron accumulation, lipid peroxidation, and oxidation of polyunsaturated fatty acid-containing phospholipids (PUFA-PLs) [103]. The presence of iron in the cytosol causes the formation of ROS through the Fenton reaction, by which H_2_O_2_ is transformed in hydroxyl radicals that, in turn, cause lipid peroxidation to play an essential role in ferroptosis [103].

ROS increase can be modulated by the quenching action of GPX4, an enzyme converting reduced glutathione (GSH) into glutathione disulphide (GSSG) that utilizes hydrogen ions to reduce hydrogen peroxide as well as lipid peroxides [104]. GPX4 can be therefore considered a type of GSH peroxidase, representing the main intracellular antioxidant enzyme acting against ROS. In fact, it prevents iron dependent formation and accumulation of ROS, producing lipid alcohols (R–OH) from lipid hydroperoxide (R–OOH) with GSH as a cofactor. This metabolic process is cytoprotective against Fe^2+^-dependent formation and accumulation of ROS [103,105].

Beside the central role of GPX4 in maintaining ROS low levels, a corollary of different pathways is necessary to support the efficiency of GPX antioxidant activity. First, the so-called System Xc^−^, a heterodimeric antiport system that imports cystine and exports glutamate, formed by two subunits named SLC3A2 and SLC7A11 [106]. The imported cystine is further transformed in cysteine and then into GSH, in order to maintain redox homeostasis thus protecting cells from ferroptosis [107]. System Xc^−^ is the main mechanism supporting the efficiency of GPX antioxidant activity through the inhibition of a series of events triggering the reduction of GSH levels, lipid peroxidation, and consequent ferroptosis [103].

The second pathway that cooperates with GPX is composed of a protein complex involved in the enzymatic cascade that leads to the hydro peroxidation of lipid-polyunsaturated fatty acids (PL-PUFA-OOH), which represent the substrate for GPX4 to be oxidized and transformed into lipid-polyunsaturated fatty acids alcohols (PL-PUFA-OH). Several enzymes involved in the production of PL-PUFA-OOH are important in ferroptosis processes due to their regulation being associated with this particular type of cell death, namely ACSL4, LPCAT3, LOX [108].

**Figure 4:**
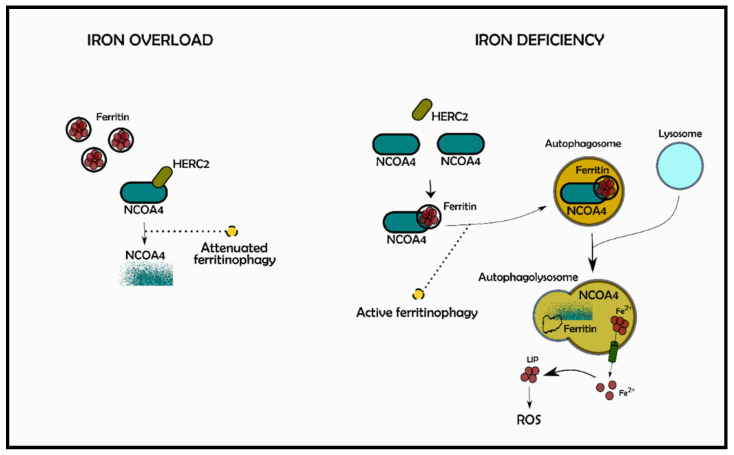
NCOA4-mediated *ferritinophagy*. Intracellular iron is regulated by a process known as ferritinophagy. In conditions of iron overload (on the **left**), NCOA4 is degraded by the binding with HERC2, an ubiquitin ligase protein, leading to the reduction in ferritin degradation. On the contrary, during iron deficiency (on the **right**), NCOA4 molecules stabilize being able to bind ferritin, thus activating ferritinophagy via lysosomal degradation. NCOA4: Nuclear Receptor Coactivator 4; HERC2: HECT and RLD domain Containing E3 Ubiquitin Protein Ligase 2; LIP: Labile Iron Pool.

In this particular case, ferroptosis depends on reduced detoxification of lipid peroxides through the action of the enzymatic activity of GPX4 [109] and, most recently, it was reported that its sensitivity can be also regulated by Nuclear Receptor Coactivator 4 (NCOA4) [110], a cargo protein involved in autophagic ferritin degradation [111]. Cytosolic ferritins work as iron storing molecules and are composed of 24 subunits of H (heavy) and L (light) chains that co-assemble in different proportion to form heteropolymers with a tissue specific distribution combined to functions: Ft heteropolymers present in organs designated to iron storage, as liver and spleen, are richer in L-subunits, while those present in heart and brain, that have a high ferroxidase activity, are richer in H-subunits [112]. The FtH subunit has enzymatic activity and oxidizes Fe^2+^ to Fe^3+^ to be incorporated into the protein shell. As a whole, Ft polymers are involved in iron detoxification, storage, and recycling [112]. The upregulation of ferritin can limit ferroptosis; the opposite effect occurs if the protein is downregulated [113]. The binding between NCOA4 and ferritin is FtH specific [114] and, as a consequence of it, FtH is carried into lysosomes, degraded, and iron is released to be used by the cells, modulating in this way the intracellular iron amount, in a process termed “ferritinophagy” [111,113]. Consistently, NCOA4 levels are regulated by intracellular iron status [115] and recent works showed that the amount of NCOA4 changes according to the interaction with an E3 ubiquitin protein ligase, HERC2 [115,116]; during iron overload condition, NCOA4 is degraded by HERC2, whose activation is in turn iron dependent (Figure 4) [113].

Since intracellular iron regulation depends on NCOA4-mediated ferritinophagy, the lack of NCOA4 in a murine model induced systemic iron overload congruent to increased level of transferrin saturation, serum ferritin, liver hepcidin and ferritin deposits with decreased iron availability [117].

It is known that ferroptosis is a form of autophagic cell death [118]; autophagy promotes ferroptosis leading to ferritin degradation via ferritinophagy mediated by NCOA4 with an increase of labile iron pool (LIP), which promotes ROS accumulation, the main driver of the ferroptotic mechanism [118]. On the other hand, NCOA4 inhibition limits ferritin degradation and blocks the ferroptosis process; the opposite effect is triggered if NCAO4 is overexpressed [113].

The activation of cell defence mechanisms which enhance the detoxification of cells is also mediated by Nrf2 [119]. Nrf2 is a redox-sensitive transcription factor, whose activation results in cellular antioxidant responses modulating several stress-responsive proteins and phase II detoxification [119,120]. In normal conditions, Keap1 stimulates Nrf2 ubiquitination and its proteasomal degradation in order to keep its levels low; on the contrary under oxidative stress condition, Nrf2 disassociates from Keap1, translocates to the nucleus where it binds to antioxidant response element (ARE) located in the promoter region of target genes, and regulates/activates the transcription of heme oxygenase-1 (HO-1), NAD(P)H, quinone oxidoreductase (NQO1), glutathione S-transferase (GST), and glutathione peroxidase (GPx), favouring several cellular defence mechanisms and amplifying detoxification [119,121]. Nrf2 works as a ferroptosis suppressor since it promotes the expression of antioxidants or iron metabolism genes involved in iron import, as Transferrin receptor 1 (Tfr1) and iron storage protein ferritin [112,122]. The perturbation in iron metabolism during the process of import, export and storage of iron may influence the cell hypersensitivity to ferroptosis. Data has shown that pseudolaric acid B (PAB) treatment of glioma cells causes upregulation of Tfr1 and the resulting increased iron entry into the cells activates Nox4 leading to production of H_2_O_2_ and lipid peroxides, all signs of ferroptotic process activation, [123]. Following the same rationale, using prominin2, a pentaspan protein involved in regulation of lipid metabolism, in mammary epithelial and breast carcinoma cells, improves ferroptosis resistance by promoting ferritin export [124].

It has been demonstrated that both the Nrf2 genetic inactivation in knockdown mouse Hepa1-6 and its pharmacological inhibition using erastin and sorafenib, trigger ferroptosis events in vivo [122]. This confirms that the loss of Nrf2 improves the ferroptosis process through the consequent reduction of the anti-ferroptosis gene transcription including FtH, HO-1, and NADPH quinone oxide-reductase, EC. 1.6.99.2 (NQO1) [122]. Furthermore, Nrf2 is able to upregulate a large number of other anti-ferroptotic genes such as glutamate–cysteine ligase catalytic subunit (GCLC), glutathione synthetase (GSS) and GPX4 to enhance GSH synthesis and function [125].

In this context, the chaperones protein HSB1 (Heat Shock protein Beta-1), protects cells against harmful stimuli [126]. It has also been demonstrated that, after erastin treatment, HSB1 is upregulated to inhibit ferroptosis by reducing cellular iron uptake through the inhibition of Tfr1 and lipid ROS production, in different cancer cells [127].

## 9. Conclusions

Similar to many other elements, iron is both detrimental and indispensable for life. The subtle balance between too low and too much iron, is regulated at the level of both the entire organism and the single cell. Moreover, iron continuously changing between the reduced and oxidised forms, adds complexity to its trafficking between the intra- and extracellular compartments because selective carriers are needed for its transport even inside cell organelles [128]. Perhaps the best model to study the mechanisms for iron import and export, are the enterocytes where iron assumed with the diet, is both internalised and externalised in the apical and basolateral membrane, respectively [129]. Nevertheless, it is important to unravel how every organ is able to manage and respond to iron variation to minimize the adverse effects that iron overload or deficiency could cause in that specific compartment. A typical example is represented by the central nervous system that, even in the condition of systemic anemia, maintains constant its own iron quantity [130]. As for iron overload, independent of the cause, it triggers detrimental processes within cells and the type of cell death induced by iron accumulation has been named ferroptosis [131]. While it is true that all cell types are affected by iron unbalance, two organs are paradigmatic in the ferroptosis scenario; the heart is one of the principal utilisers of iron while the liver is principally responsible for iron handling. Iron level alterations are detrimental in both directions as proved by the poor prognosis of heart failure patients with commonly associated iron deficiency [132]. Accordingly, iron mishandling has been proposed to be a major trigger for severe injury to cardiomyocytes and hepatocytes and therefore an important player in developing heart and liver failure [55,133]. On the other hand, while iron overload has been demonstrated as being associated with several cardiac diseases, its essential role in mediating ischemic preconditioning cardioprotection has been proposed [55]. These reports are in accordance with studies demonstrating the double role of ROS in mediating both injury and protection to the heart, ROS production being strictly related to iron activity inside the cell [134]. With the surge of results confirming the pivotal role of ferroptosis in developing heart and liver diseases, some recent studies have demonstrated how targeting the ferroptotic pathway can be beneficial in inhibiting cell death induced by iron overload. For instance, the use of both iron chelator and antioxidants have been proposed as therapeutic tools in the treatment of myocardial infarction and in preconditioning the hearts of patients undergoing reperfusion manoeuvres [135]. In liver, several physiopathological pathways can be affected by impaired iron homeostasis including alcohol-induced hepatotoxicity [136], acute liver failure [85], drug-induced liver injury [137], and hepatic fibrosis [138]. Thus, clarifying the subtle mechanisms that regulate iron balance within hepatocytes is of paramount importance in the attempt to mitigate the clinical outcome of the above-mentioned pathologies.

Although the main focus of this review is cardiac and hepatic ferroptosis, it is worth to briefly mention that ferroptosis emerged as a pivotal and broad mechanism responsible for cell death occurring in several other cell types. Consistently, ferroptotic cell death has been described as being implicated in a broad spectrum of disorders including, but not limited to, cancer, diabetes, acute kidney injuries and neurodegenerative disorders, and sometimes also associated with the progression of diseases like leukaemia, psoriasis, and haemolytic disorders [139].

## Figures and Tables

**Table 1:** List of the main genes submitted to IRE/IRP post-transcriptional regulation. Localization of the IRE element (5′ or 3′ untranslated regions (UTRs)) is reported for each gene mRNA; arrow down indicates the translation block caused by the IRPs binding to IRE sequence and diminished effect, arrow up indicates mRNA stabilization due to the IRPs binding to IRE sequence and augmented effect.

RNA	Protein	5′UTR	3′UTR	Translation	Effect	Refs.
**FtH/L**	Ferritin	√		↓	↓ iron storage protein	[17]
**DMT1**	Divalen Metal Transpoter		√	↑	↑ Iron import	[17]
**TfR1**	Transferrin Receptor		√	↑	↑ Iron import	[17]
**Fpn1**	Ferropotin 1	√		↓	↑ Iron export	[17]
**ACO2**	Mitochondrial Aconitise 2	√		↓	↓ TCA cycle	[17]
**HIF2α**	Hypoxia-Inducible Factor 2α	√		↓	↓ Hypoxia reponse	[17]
**ALAS2**	△-Aminolevulinate Synthase 2	√		↓	↓ Heme biosynthesis	[17]
**CDC14A**	Cell cycle phosphatase var.1		√	↑	-	[18]
**CDC42BPA**	Myotonic dystrophy kinase-related Cdc42-binding kinase α		√	↑	↑ Tfr1	[19]
